# Cyclically sheared colloidal gels: structural change and delayed failure time

**DOI:** 10.1039/d5sm00647c

**Published:** 2025-10-23

**Authors:** Himangsu Bhaumik, James E. Hallett, Tanniemola B. Liverpool, Robert L. Jack, C. Patrick Royall

**Affiliations:** a Yusuf Hamied Department of Chemistry, University of Cambridge Lensfield Road Cambridge CB2 1EW UK; b Department of Chemistry, School of Chemistry, Food and Pharmacy, University of Reading Reading RG6 6AD UK; c School of Mathematics, University of Bristol Fry Building Bristol BS8 1UG UK; d DAMTP, Centre for Mathematical Sciences, University of Cambridge Wilberforce Road Cambridge CB3 0WA UK; e Gulliver UMR CNRS 7083, ESPCI Paris, Université PSL 75005 Paris France

## Abstract

We present experiments and simulations on cyclically sheared colloidal gels, and probe their behaviour on several different length scales. For experimental gels formed by colloid–polymer mixtures, the shearing induces structural changes, which are quantified by the topological cluster classification, bond-order parameters, and the pore size distribution. These results are mirrored in computer simulations of a model gel-former: for cyclic shear with amplitudes up to 4%, local structural analysis shows that the material evolves down the energy landscape under shearing, and the average pore size increases. We also analyze mechanical responses including the stress and the dissipation rate, revealing a crossover between elastic and plastic responses as the strain amplitude is increased. Depending on the parameters, we observe both increased compliance after shearing (thixotropy), and reduced compliance (strain hardening). We simulate creeping flow under constant shear stress, for gels that were previously subject to cyclic shear, showing that strain-hardening also increases gel stability. This response depends on the orientation of the applied shear stress, revealing that the cyclic shear imprints anisotropic structural features into the gel.

## Abstract 2

Experiments and simulations show how cyclic shearing affects the structure and dynamics of colloidal gels.

## Graphical abstract

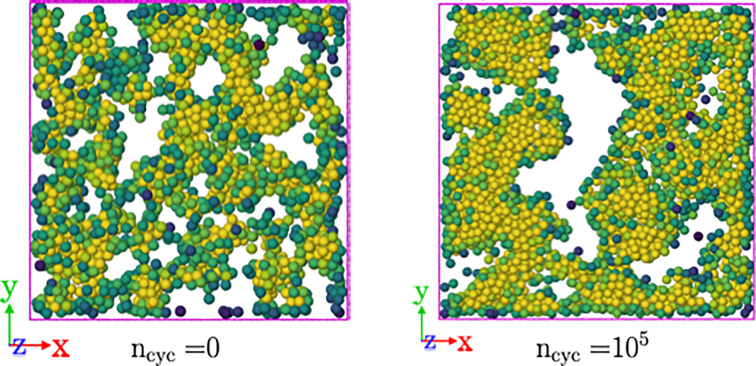

## 1. Introduction

Colloidal gels are assemblies of particles consisting of interconnected networks of strands, which are kinetically arrested far from equilibrium.^1–6^ They exhibit complex relaxations and responses to external forces, whose understanding is important for applications, and as a fundamental outstanding problem in soft matter.^7–11^ These materials are particularly challenging because their behaviour is influenced by structural features on different length scales, including local motifs in the microscopic structure,^12^ the response of individual strands to applied stress,^13–15^ and the heterogeneous network.^16–20^

Gel structure depends on particle interactions (strength and range of attractive forces) and on their volume fraction. In addition, the fact that gels are far from equilibrium means that their structures also depend on their mechanical and thermal history; they also experience physical aging, so their properties depend on the time elapsed since their preparation.^21–29^ This feature can be exploited to engineer materials with specific properties, but the relationships between history, structure and gel properties are complex, and theoretical predictions are limited, so that formulation of gels often requires a large component of trial-and-error.

Among the material properties that one would like to control are the linear response to external stress (compliance) and the yielding behavior.^30,31^ The process of strain-hardening offers a promising route for this, in that mechanical processing of an already-formulated material can be used to suppress yielding and/or reduce compliance. Strain-hardening is familiar in other amorphous solids such as molecular and metallic glasses^32–34^ where the shear provides a mechanism for the system to descend in its energy landscape and form more stable structures (see also ref. 35). Shear-thinning can also occur in glasses^36^ and the rheology of glasses and dense suspensions is a rich topic in its own right.^37,38^

In colloidal gels, the underlying network structure makes the behaviour even richer than glassy or non-Brownian suspensions. The pioneering work of Gisler *et al.*^39^ showed evidence for strain-hardening in fractal colloidal gels, see also ref. 40–42; disruption of the network by shearing is a mechanism for shear-thinning (thixotropy)^30,43^ as also found in theoretical and computational models.^44–46^ Studies on a wide range of experimental colloidal gels show that indeed mechanical history affects the subsequent rheology, with examples including colloid–polymer mixtures,^47–51^ carbon black,^52,53^ silica,^54^ and clays.^55–57^ A well-characterised example of such history dependence is the Bauschinger effect,^43^ in which the direction of an applied shear is reversed; the subsequent (“backward”) response retains a dependence on the original (“forward”) shear strain.^58^ More recent works have used history-dependence to imprint more complex memories in gel structure,^59^ building on previous work on athermal systems,^60,61^ see also ref. 62. The extent to which shear flow is able to break bonds in the gel structure can be quantified by the Mason number,^63,64^ which determines the relative strength of the applied shear stress and the interparticle forces (see also ref. 47).

The systems considered here are gels that form by depletion in colloid–polymer mixtures, which we analyse both experimentally and by computer simulation. The experiments combine a shear stage with *in situ* particle-resolved imaging by 3d confocal microscopy, enabling microscopic changes in structure to be probed. Such methods have been used extensively to investigate colloidal glasses.^65–67^ In the case of colloidal gels, combined imaging and shear has enabled direct observation of shear induced microstructural changes.^51,68,69^ Rheological methods such as cyclic shear can also be used to identify a gel point where the elastic and viscous moduli cross.^70^ The depletion interactions in these systems lead to interparticle attractions that are comparable to the thermal energy; this may be contrasted with the stronger interactions found in carbon black, or systems dominated by van der Waals interactions. Nevertheless, depletion can produce stable gels, whose properties can be further tuned by their mechanical history, including strengthening *via* rejuvenation.^48,50^

We briefly summarise our results, before proceeding to a more detailed discussion. We first measure structural changes under cyclic shear, using both experiments and simulations. The gel structures evolve under shear by two important processes: the breaking of gel strands,^13–15^ and the evolution of the microscopic structure towards lower energies.^71^ The experimental data reveal this through their pore-size distributions (mesoscopic structure) and through the topological cluster classification (TCC)^72^ which characterises the microscopic structure. The same behavior is also present in our computer simulations.

The second set of results shows the dynamical behavior of gels in computer simulation, including the rheological properties during cyclic shear, and measurements of the yielding under constant stress (creep). We present evidence for strain hardening, based on compliance and dissipation during the shear cycles. We also subject the resulting gels to constant stress, which leads to creeping flow and eventual yielding. These samples survive for longer times before failure, compared to gels that were not strain-hardened. Moreover, this enhanced stability depends significantly on the relative directions of the shearing motions for the cyclic and creeping protocols. In other words, the hardening can be used to enhance gel stability and to imprint anisotropic responses to subsequent shearing.

These results demonstrate specific situations in which gel properties can be selected by mechanical processing. The particle-resolved experiments reveal the microscopic and mesoscopic changes in structure; the simulations show how these can be harnessed to enhance gel stability and to tailor anisotropic responses. We discuss how our understanding of these far from equilibrium materials might be harnessed to for prediction and design of material properties more generally.

The structure of the paper is as follows: Section 2 describes models and methods, and Section 3 presents results for structural change due to cyclic shear. Section 4 discusses the dynamical behaviour, after which Section 5 concludes with a discussion of our results and directions for future work.

## 2. Models and methods

We consider gels formed of spherical colloidal particles with attractive interactions, leading to reversible bonds between them. These bonds may be strong enough that the suspension supports dense (colloidal liquid) and dilute (colloidal vapour) phases. In this case gels form by arrested spinodal decomposition,^3,5^ whose dynamical arrest is controlled by the colloidal liquid phase where the packing is dense, particles have many bonds, and the local structure is reminiscent of colloidal glasses,^5,73^ see also ref. 74. Due to the link with spinodal decomposition,^3^ the effective attraction at the onset of gelation is very close to its value at the colloidal liquid-vapour critical point, which may be related to the microscopic interaction potential by the Noro–Frenkel criterion.^75^

We briefly summarise the system's control parameters, with further details given below. To characterise the effective interactions in the simulations and experiments we consider the ratio *q* between interaction range and the colloid diameter *ℓ*; and the ratio *ε*_0_ = *ε*/(*k*_B_*T*) between effective interaction strength *ε* and the thermal energy. The colloid volume fraction is *ϕ*. For systems under cyclic shear, the shear amplitude *γ*_0_ is a relevant dimensionless parameter, as is the product *γ̇τ*_B_ of the shear rate with the Brownian time *τ*_B_ = π*η*_s_*ℓ*^3^/(8*k*_B_*T*), where *η*_s_ is the solvent viscosity. The Mason number is proportional to *γ̇τ*_B_/*ε*_0_, the relevance of this number to our cyclic shear experiments is discussed in Section 5.

### 2.1. Experiment

#### 2.1.1. Colloidal particles and effective interactions

We consider sterically stablised poly-methyl methacrylate colloids in a *cis*-decalin and cyclohexylbromide solvent mixture which matches the density and refractive index. The particles were fluorescently labelled with 1,1-dioctadecyl-3,3,3,3-tetramethyl-indocarbocyanine perchlorate.^76^

The particles are dispersed in a solution (4 mM) of tetrabutyl ammonium bromide, to screen the electrostatic interactions. Depletion attractions are provided by a nonadsorbing polymer (polystyrene, molecular weight 8.4 MDa). The (mean) particle diameter is *ℓ* = 1500 nm, we estimate the polydispersity at 5%. We estimate the Brownian time as *τ*_B_ = 4.0 s (see above for the definition). The colloid volume fractions are obtained from the imaging results, see Table 1.

**Table 1:** Experimental parameters for shear. The rate *γ̇* is given relative to the Brownian time

*ε*	*γ̇* (/*τ*_B_^−1^)	*γ*_max_	*ϕ*
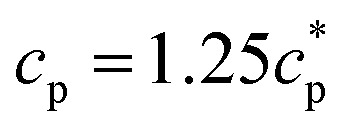	3.72	0.0337	0.17
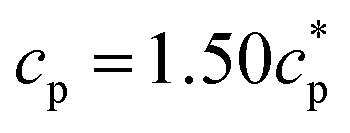	9.32	0.0337	0.18

Our experiments at these volume fractions show that the onset of gelation occurs at concentration 
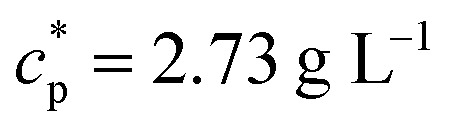
. Here we present data for polymer concentration *c*_p_ in the range 3.42–4.10 g L^−1^, specifically 
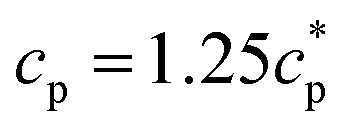
 and 
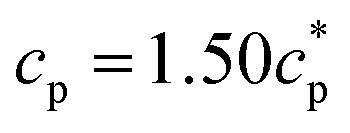
. The sample dimensions are 1 cm × 1 cm × 100 μm. We extract the positions of the particles from confocal microscopy images using the trackpy package. We consider the particles inside a cubic visualisation region (“box”) of linear size *L* ≈ 33*ℓ*. This results in co-ordinates of *N* ≈ 1.5 × 10^4^ particles, which show that the particles form a percolating network, characteristic of the gel state.

To estimate more precisely the effective interactions, we follow previous work^73^ by inferring the parameters of an one-component Asakura-Oosawa (AO) model,^77^ which is known to be a reasonably accurate description of colloid–polymer mixtures.^78–80^ In addition to the particle diameter *ℓ*, its parameters are the polymer radius of gyration *R*_g_ and the polymer fugacity *z*_p_ (proportional to *c*_p_, which is the “reservoir” concentration^73^).

As discussed in ref. 73, estimating *R*_g_ directly from properties of the polymer and solvent leads to relative errors around 10%, which are significant because the interaction strength varies as *R*_g_^3^.^77^ Instead we infer AO parameters consistent with the experiment by assuming that the onset of gelation at 
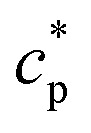
 coincides with the polymer concentration at the liquid–vapor critical point of the AO model; this can be related to the underlying interaction potential by the Noro–Frenkel extended law of corresponding states.^75^ (This approach for inferring interactions is effective in practice;^73^ it may justified by the fact that gelation in colloid–polymer mixtures proceeds by arrested spinodal decomposition,^3,5^ note also that the onset of gelation 
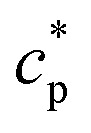
 is weakly dependent on the colloid volume fraction *ϕ* in the range considered here.)

This approach yields a polymer radius of gyration *R*_g_ = 155 nm, which is a consistent estimate for this polystyrene polymer, in a good solvent; the depth of the attractive potential well at the critical point is ε* = 3.16*k*_B_*T*. Hence the polymer–colloid size ratio is *q* = (2*R*_g_/*ℓ*) = 0.207. Note that for this size ratio, the colloidal liquid–vapour critical point is metastable to fluid-crystal phase coexistence.^6^ Since *ε* ∝ *c*_p_ the two polymer concentrations considered here then correspond to dimensionless interaction strengths *ε*_0_ = *ε*/(*k*_B_*T*) = 3.95 and 4.74.

#### 2.1.2. Shear cell and shearing protocol

We use a shear stage to apply cyclic (simple) shear with motion in the *y* direction and velocity gradient in the *z* direction. The shear stage comprised an aluminium flexure with a coverslip window, mounted on a plexiglass baseplate and separated by a rubber spacer, which served to both dictate the gap thickness and to prevent solvent evaporation. Both the top coverslip and bottom glass surface were coated with a sintered particle layer to allow the gel to adhere to both surfaces. Displacement of the top window was driven by a Thorlabs TPZ001 T-cube piezo driver connected to a Thorlabs piezo stack with free stroke displacement of 25.5 microns, controlled by a MATLAB script.

The shear is a triangular wave with constant rate *γ̇*, that is, the strain is increased linearly in time from *γ* = 0 to *γ* = *γ*_max_ and then decreased to *γ* = −*γ*_max_ before finally increasing back to *γ* = 0. We alternate shearing and imaging, the acquisition time for an 3d image of the gel is approximately 30 s (7.5*τ*_B_); there is negligible aging during this time. The location of the visualisation region does not stay constant with time, this ensures that data is not too much affected by photobleaching that would occur if all imaging occurred in the same place. The waiting time *t*_w_ between sample loading and the start of shearing is approximately 30 minutes (450*τ*_B_).

We consider two state points that differ in the polymer concentration and the parameters of the applied shear (details given above). The parameters for the shear experiments are reported in Table 1.

### 2.2. Computational

#### 2.2.1. Model

For numerical simulations, we use an established model of a size-polydisperse colloid–polymer mixture^9,12,24,80–82^ which has been accurately mapped to the standard two-component Asakura-Oosawa model.^77,80^ The depletion interaction between colloidal particles is modeled by a truncated and shifted Morse potential

1 *U*(*r*) = *ε*[e^−2*α*(*r−ℓ*_*ij*_)^ − 2e^−*α*(*r*−*ℓ*_*ij*_)^ + *c*_sh_], *r* ≤ *r*_c_

where *ℓ*_*ij*_ is the average diameter of particles *i* and *j*; the interaction strength is *ε* and *α* sets the range; the cutoff parameter is *r*_c_ = 1.4*ℓ*_*ij*_, and we truncate so that *U*(*r*) = 0 for *r* > *r*_c_; the constant shift *c*_sh_ is chosen so that *U* is continuous at *r* = *r*_c_. We consider *N* particles in a cubic box of volume *L*^3^, with periodic boundaries.

The overdamped colloid motion is modeled through Langevin dynamics with a large friction constant (see below for further details). Particles have mass *m* and they evolve with Langevin dynamics so the position ***r***_*i*_ of particle *i* obeys d***r***_*i*_/d*t* = ***v***_*i*_, and2
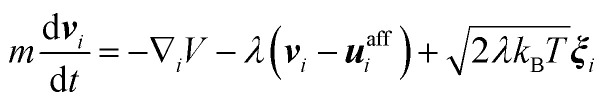
where 
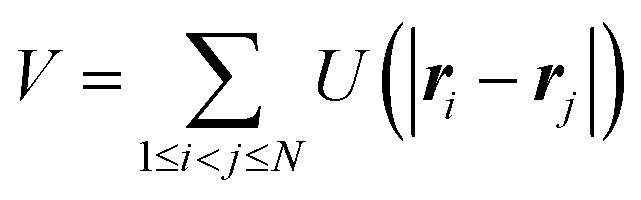
 is the total potential energy, *λ* is the friction constant, ***u***^aff^_*i*_ is the local velocity of the (implicit) solvent, and ***ξ*** is a standard Gaussian white noise. (In the absence of shear flow ***u***^aff^_*i*_ = 0, the sheared case is discussed below.) The velocity damping time is *τ*_d_ = *m*/*λ*. All simulations are performed in LAMMPS.^83^

To avoid crystallization we consider a size polydisperse system. We have taken 7 types of particles with diameters equally spaced between 
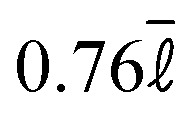
 and 
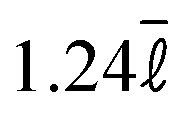
, with relative concentrations [0.0062,0.0606,0.2417,0.3829,0.2417,0.0606,0.0062] to mimic a Gaussian distribution of diameters with 8% polydispersity.

We work with non-dimensionalized parameters throughout. As in ref. 84, we estimate the critical interaction strength for spinodal decomposition as ε* ≈ 3.1*k*_B_*T*, based on the Noro–Frenkel criterion.^75^ We report interaction strengths relative to this boundary, we focus on two state points with *ε* = 4.5*k*_B_*T* = 1.45ε* and *ε* = 10*k*_B_*T* = 3.22ε*. Our unit of distance is the mean particle diameter 
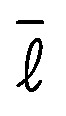
 and the unit of time is 
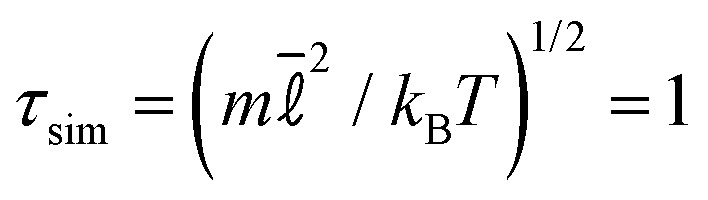
. The colloid volume fraction is 
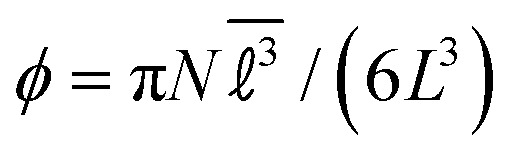
. The dimensionless parameter governing the interaction range is 
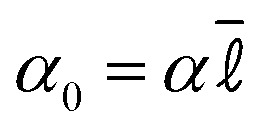
, we set *α*_0_ = 33 throughout. The non-dimensionalised friction is *λ*_0_ = *λτ*_sim_/*m*. We take *λ*_0_ = 10 to mimic overdamped dynamics, larger values of *λ*_0_ would approximate more accurately colloidal motion in the solvent but the computational cost is higher and we have found previously that it changes very little the emergent behaviour.^81^ The integration time step is Δ*t* = 0.001*τ*_sim_. A natural time scale for colloid motion is the Brownian time 
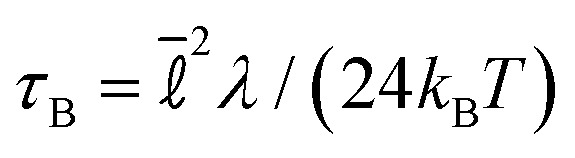
 which is the typical time for an overdamped free particle to diffuse its radius. For the parameters chosen here *τ*_B_ = 0.417*τ*_sim_.

This simulation method is computationally efficient and has been shown to capture the essential features of depletion gels,^9,24^ including quantitative comparisons with experiment.^12,81^ As such, it complements modelling approaches that focus on the gels' network structure^44^ but do not resolve the internal structure of the gel strands. On the other hand, our model neglects hydrodynamic interactions, which do affect some aspects of gel structure.^85–88^

In the following, we have not attempted a quantitative matching of the parameters of simulation and experiment, because the implementation of the cyclic shear is necessarily different (not least that the simulation uses periodic boundary conditions, and an idealized thermostat that has to absorb the dissipated heat during the shear but has not been parameterised for the experiment). Despite these differences, we will show that the simulations and experiments exhibit the same qualitative behaviour.

#### 2.2.2. Simulations

##### 2.2.2.1. Initialisation

Simulations are initialised in random configurations at volume fraction *ϕ* = 0.2. Gels are formed by simulating for a time *t*_w_, during which spinodal decomposition occurs. Unless otherwise stated we simulate *t*_w_ = 3 × 10^4^*τ*_sim_ (≈7 × 10^4^*τ*_B_) and system size of *N* = 10^4^ particles; all results are averaged over many independent runs (typically 50), to enable statistically robust conclusions.

##### 2.2.2.2. Cyclic shear

After gel preparation we perform strain-controlled cyclic shear at finite temperatures and strain rates. The strain *γ*_*xy*_ = *γ*(*t*) of the system is varied cyclically with a triangular wave with amplitude *γ*_max_ and rate |*γ̇*| = 0.01*τ*_sim_^−1^. This cycle is repeated many times. The simulated shear rates are significantly slower than those of the experiment, to ensure that the thermostat can easily absorb the energy that is injected by this external work. We would expect qualitatively similar behaviour for larger shear rates. We compute observable quantities at the stroboscopic configuration (*γ* = 0) after each cycle of strain and study the evolution of these quantities as a function of number of cycles *n*_cyc_.

We use Lees–Edwards boundary conditions. The solvent velocity ***u***^aff^_*i*_ in (2) points in the *x* direction, with magnitude *y*_*i*_*γ̇*, where *y*_*i*_ denotes the *y*-coordinate of particle *i*.

##### 2.2.2.3. Creep simulation

Gels are non-equilibrium states and the cyclic shear affects their structure. After shearing, we performed stress-controlled creep dynamics to probe gels' yielding behaviour. The procedure follows that of ref. 84, a non-dimensionalized stress 
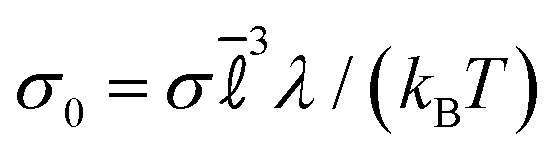
 is maintained through a feedback control scheme that is implemented^89,90^ as

3 ∂_*t*_*γ̇* = *B*[*σ*_0_ − *σ*_*xy*_(*t*)]

where *γ̇* is the shear rate and *σ*_*xy*_ is the observed (non-dimensional) shear stress [measured from the virial]; also *B* is the damping parameter determining how quickly the applied stress relaxes to its imposed value. Similar to the cyclic shear, we used Lees–Edwards boundary conditions with affine flow in the *x*-direction. We take *B* = 0.01*τ*_sim_^−2^ as in ref. 84 which ensures that the stress is imposed accurately, at a reasonable computational cost (such schemes involve inertial effects including ringing, see ref. 84 and 91 for further discussion).

## 3. Results – gel structure (experiment and simulation)

### 3.1. Gel visualisation

**Fig. 1:**
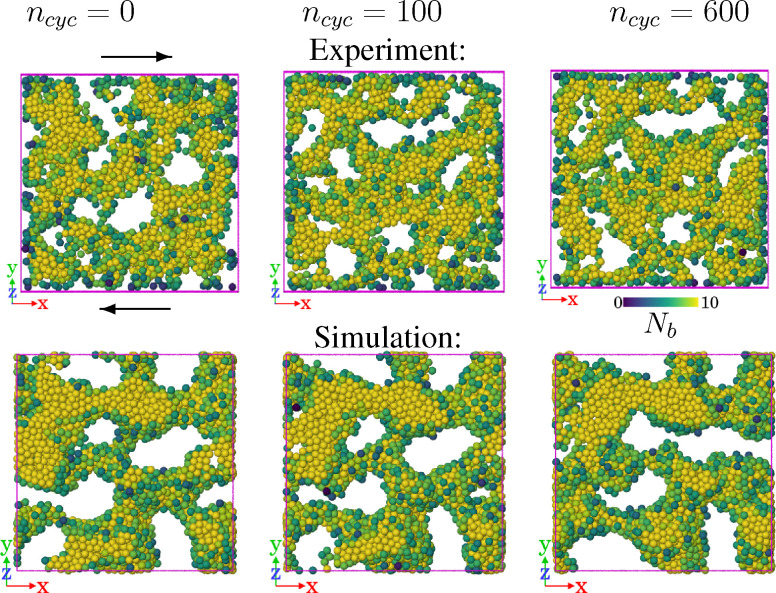
Visualization of gel under cyclic shear (experiment and simulation). (Top) Rendering of a slice through an experimental gel, to visualize the strands and pores after different strain cycles *n*_cyc_ = 0, 100, 600 for the gel with 
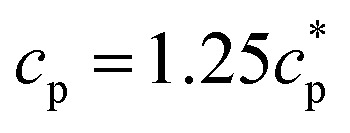
. Arrows indicate the geometry of the (simple) shear. The region shown has volume *L* × *L* × *Z* with *Z* = 8*ℓ*. (Bottom) Similar slices based on simulation data for *ε*_0_ = 1.5ε*, *γ*_max_ = 0.04. Particles are coloured by their coordination number, according to the colorbar.

Fig. 1(top) shows slices through the experimental system. (Specifically, we visualise particles in a region of size *L* × *L* × *Z* with *Z* = 8*ℓ*, for various *n*_cyc_.) One clearly sees a percolating network of colloidal particles, characteristic of the gel state. Shearing the sample for 100–600 cycles causes structural rearrangements including some pores changing their shapes. Fig. 1(bottom) presents similar slices from numerical simulation. As noted above, we have not attempted to match the parameters precisely, in particular the waiting times before start of shear are significantly longer in the simulation (leading to thicker gel strands), and the dimensionless shear rate (*γ̇τ*_B_) is much smaller in the simulations. Even so, the shearing leads to similar structural changes in both samples, in that the structure and pores are rearranged.

In the following subsections we analyse the results in detail for both experiment and simulation. We mainly focus on the structural change at different length scales due to cyclic shear deformation. From the simulations, we also show in Section 4 that the cyclic shear hardens the gel.

### 3.2. Experiment

#### 3.2.1. Microscopic structure

To probe microscopic structural changes, we measure several local quantities, which mirror those measured in the simulation study of ref. 84. Specifically, we consider the coordination number *N*_b_, the two-fold bond-orientation parameter *q*_2_, and the average number of different types of clusters in which particles participate, as obtained from the TCC.^72^ Particles within a distance of 1.8*ℓ* of particle *i* are defined as its neighbours. (This identification method for neighbours is maintained for all the experimental data analysis.) The bond orientational order parameter *q*_*l*_ is calculated following:^92^4
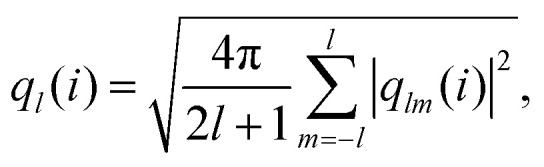
5
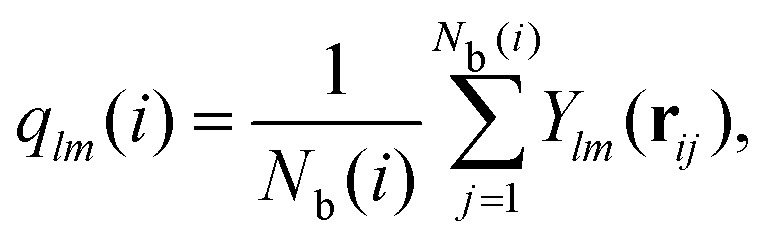
where *Y*_*lm*_ are spherical harmonics and *N*_b_(*i*) is the number of neighbours of reference particle *i*. We consider the case *l* = 2: the quantity *q*_2_ is large when a particle's bonds are collinear, which has previously been observed to correlate with bond stretching.^10^

**Fig. 2:**
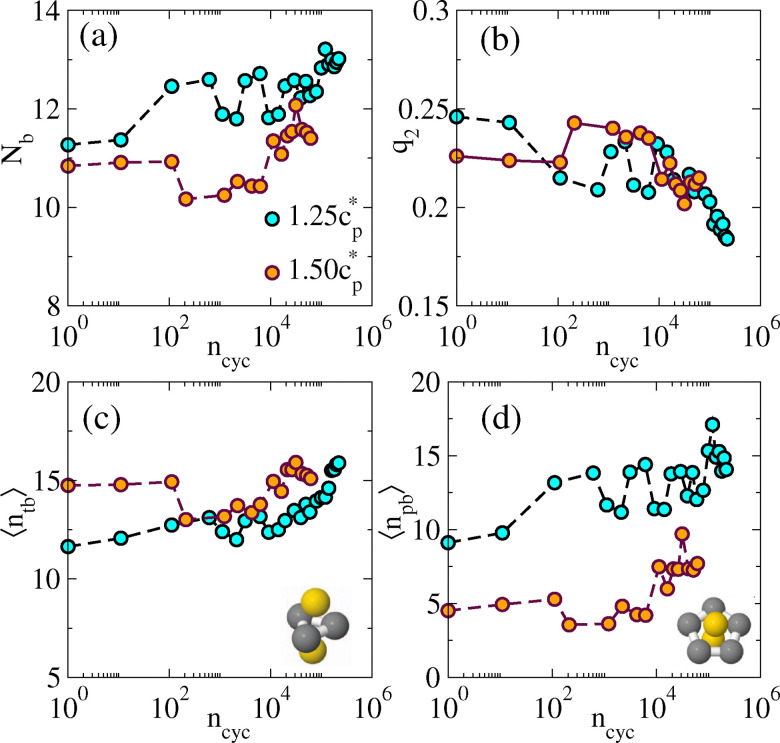
Microscopic structural analysis of sheared gel (experiment). (a) Coordination number *N*_b_ as a function of the number of shear cycles *n*_cyc_. (b) Two-fold bond orientation order parameter. (c and d) TCC analysis, showing the number of trigonal bipyramids and pentagonal bipyramids in which the particles participate. Insets show the relevant clusters. See Fig. 4 for a similar analysis of simulation data.

In Fig. 2, we show the evolution of these quantities as the system is sheared for two different gel samples. On increasing *n*_cyc_, the average coordination number *N*_b_ increases [Fig. 2(a)]. This is due to a coarsening effect whereby the shearing increases the thickness of the gel strands. Fig. 2(b) shows that *q*_2_ has a decreasing trend with *n*_cyc_. This *q*_2_ is associated with stretching of interparticle bonds: one tends to find larger values in thinner strands, and smaller values in thicker strands where the structure resembles the bulk. Hence the decreasing *q*_2_ is consistent with the increasing *N*_b_ due to coarsening.

The data show significant statistical fluctuations (each data point is based on a single set of approximately 10^4^ particle co-ordinates). Nevertheless, the systematic trends are clear, with significant changes becoming apparent after approximately 10^3^–10^4^ cycles.

We also performed a TCC analysis^72^ which detects cluster motifs as a sensitive probe of local structure. (We consider here clusters which minimize the energy for the Morse potential.) This provides a characterization of the pathway that the system makes through the free energy landscape, at a local level. Amorphous states with more bonds and denser packing tend to be rich in locally favoured structures – for spheres with short-ranged attractions these include trigonal bipyramids (5-particle clusters); pentagonal bipyramids (7-particle clusters); and icosahedra (13-particle clusters). Increasing numbers of such clusters indicate structures that are lower in the energy landscape,^12^ as happens for example during aging^71^ or annealing, see also ref. 93.

Fig. 2(c and d) show TCC results, specifically the variation of the number of trigonal bipyramids 〈*n*_tb_〉 and pentagonal bipyramids 〈*n*_pb_〉 in which a particle participates as a function of strain cycles. These TCC structures contain 5 and 7 particles respectively, and are sensitive to details of the packing. Both the quantities show an increasing trend with *n*_cyc_, indicating that the system evolves downhill in the energy landscape as the shear-induced coarsening (and aging) takes place. Similar to previous measurements, significant changes become apparent after approximately 10^3^–10^4^ cycles.

These results complement previous analysis of gels using the TCC, which considered aging,^73^ and the effects of hydrodynamics,^94^ quench rate, and polydispersity.^95^ As gelation occurs, the emergence of an elastic response is accompanied by a gel network rich in tetrahedra.^12^ As time progresses, these systems pass through a sequence of progressively largely clusters with a change from tetrahedral to fivefold symmetry,^73,95^ as annealing (or coarsening) takes place. The trigonal bipyramids considered in this work correspond to pairs of tetrahedra while the pentagonal bipyramids are larger clusters with five-fold symmetry. Hence the results Fig. 2(c and d) show that shearing that promotes annealing. (After annealing for very long times, one may expect the emergence of crystalline packings, because the colloid liquid is metastable;^6^ this effect is strongest in monodisperse systems and can be seen by the TCC.^95^ We did not see significant crystallization in this work.)

#### 3.2.2. Mesoscopic structure

We now consider the mesoscopic structure of the gel by measuring its pore size distribution. We measure the distribution of pore sizes in the gel as defined in ref. 96, the detailed procedure is described in ref. 84: We select a random point in the gel and we find the largest possible sphere that encompasses that point, without overlapping with any colloidal particles. We identify the pore size *D*_pore_ as the diameter of this largest sphere. By repeating this process for many random points we obtain the distribution of pore sizes.

**Fig. 3:**
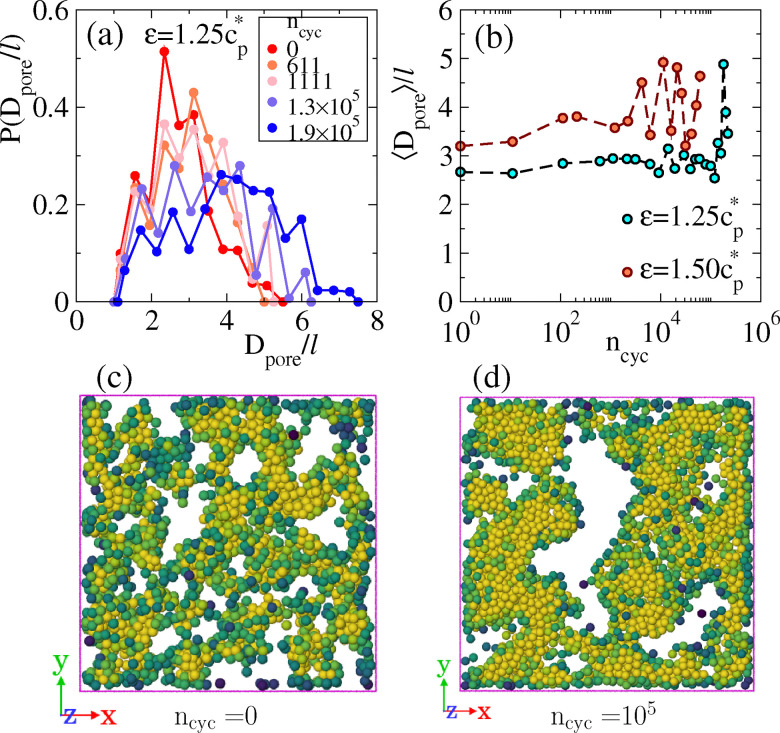
Mesoscopic (pore-size) analysis of sheared gel (experiment). (a) Distribution of pore diameter for different numbers of strain cycles for the gel with 
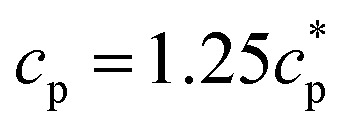
. (b) Average pore diameter against the number of cycles for two different gels. (c) Snapshot of a slice through gel before shearing 
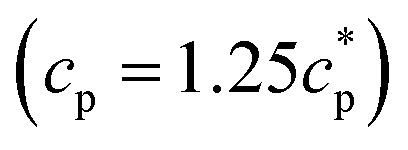
. (d) Snapshot of the same gel after 10^5^ shear cycles. Color coding in (c and d) is the same as Fig. 1. All pore-sizes *D*_pore_ are measured in units of colloid diameter *ℓ*. See Fig. 5 for a similar analysis of simulation data.

Fig. 3(a) shows the distribution of the pore sizes *D*_pore_, as the shear accumulates, for the gel with 
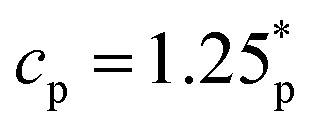
. The distribution shifts towards larger pores as the shear *n*_cyc_ increases. The maximal *D*_pore_ also has an increasing trend with *n*_cyc_. The associated mean pore size [Fig. 3(b)] has a mild initial increase before growing much more suddenly. Similar to the microstructural measurements, significant changes become apparent after approximately 10^3^–10^4^ cycles.

We find that pores can grow very large after extensive shearing, see Fig. 3(c and d) for *n*_cyc_ = 0, 10^5^ respectively. For the sample with 
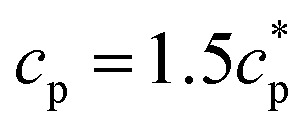
, the data of Fig. 3(b) are consistent with an increasing trend for the mean pore size, but the results are subject to large fluctuations because the data contains a small number of large pores which strongly affect the estimated average. (Recall that the location of the visualisation box is not constant in time, so the large changes as a function of *n*_cyc_ do not represent large local changes.)

### 3.3. Numerical simulation

We now analyse results of numerical simulations, and compare with experiment. We consider two state points with different interaction strengths *ε* = 1.45ε*, 3.22ε*. At the microscopic level it has been shown previously for this system^84^ that increasing *ε* during gel preparation leads to strands of reduced thickness with smaller *N*_b_ and larger *q*_2_. Here we analyse the effect of the cyclic shear.

**Fig. 4:**
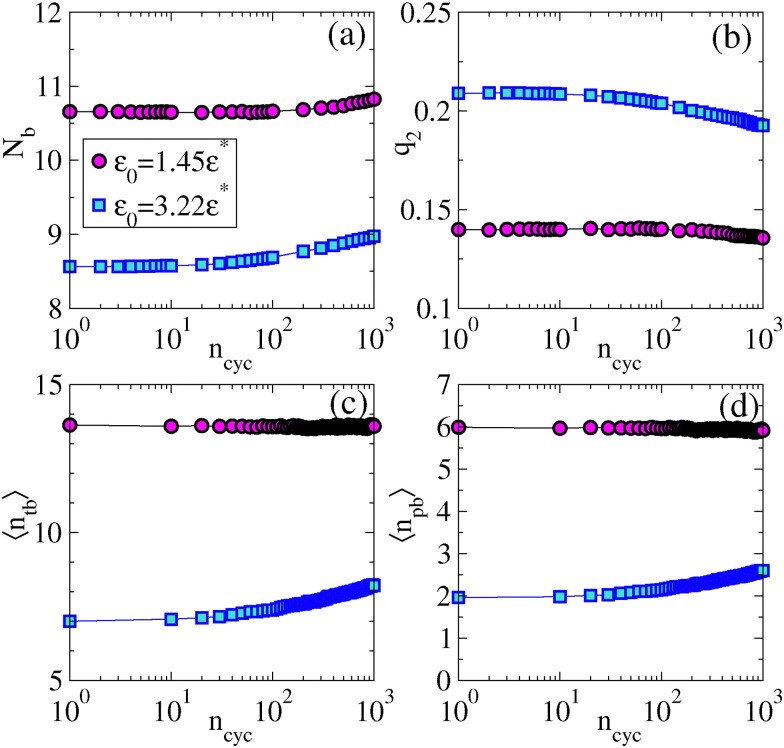
Microscopic structural analysis of sheared gel (simulation), *γ*_max_ = 0.04. (a) Coordination number *N*_b_ for increasing shear *n*_cyc_. (b) Bond orientation order parameter *q*_2_. (c and d) TCC analysis showing the number of trigonal bipyramids and pentagonal bipyramids in which the particles participate. (These results may be compared with Fig. 2.).

To study microscopic structure, particles within the interaction range *r*_c_ of particle *i* are defined as its neighbours for the evaluation of the co-ordination number *N*_b_ and for *q*_2_. We present the variation of *N*_b_ and *q*_2_ with the number of strain cycles in Fig. 4(a and b). The increasing behaviour of *N*_b_ and a decreasing trend in *q*_2_ with *n*_cyc_ mirror the experimental observations of Fig. 2. In Fig. 4(c) and (d) we present the data for 〈*n*_tb_〉 and 〈*n*_pb_〉 obtained from TCC analysis. For *ε* = 3.22ε*,we see a significant increase in the value of 〈*n*_tb_〉 and 〈*n*_pb_〉 mirroring experimental data. For *ε* = 1.45ε*, the change in these data are negligible, despite the increasing co-ordination number. This may indicate that the microscopic structure inside the gel strands is close to that of the (metastable) colloidal liquid, due to the long annealing times for the gel, and the relatively weak bonds. Then the agitation by shearing has little effect on the structure, although there is some coarsening of the strands, which increases *N*_b_.

For comparison with experiment, recall that we identify 
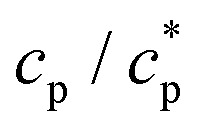
 with *ε*/ε* so that the simulation state point *ε* = 1.45ε* is comparable to the experiments of Section 2.1; the simulations with *ε* = 3.22ε* have significantly stronger interactions than the experiment. These stronger interactions promote dynamical arrest *via* kinetic trapping: this leads to thinner gel strands and local structures that are less deep in the energy landscape, consistent with the smaller *N*_b_ and reduced number of trigonal/pentagonal bipyramids.

**Fig. 5:**
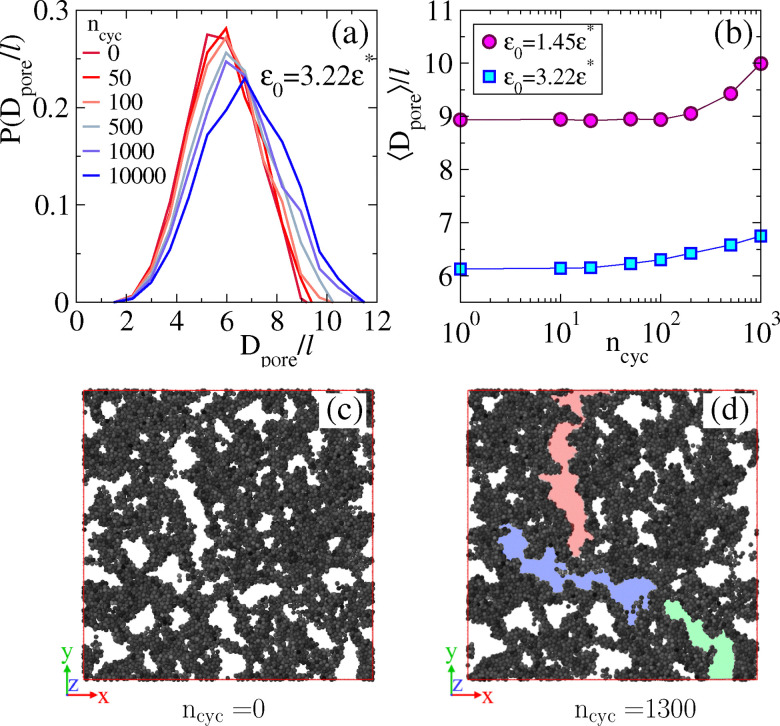
Mesoscopic (pore-size) analysis of sheared gel (simulation), *γ*_max_ = 0.04. (a) Distribution of pore diameter for different numbers of strain cycles. (b) Average pore diameter against the number of cycles. (c) Snapshot of a slice through the gel, before shearing (interaction strength *ε* = 3.22ε*). (d) The same gel after *n*_cyc_ = 1300 shear cycles; three different elongated pores are highlighted with different colours. (These results may be compared with Fig. 3 where one large elongated pore is also visible.).

Next, we investigate the mesoscopic structure of the system by analyzing the pore size of the gel. As above, we extract the distribution of the pore size *D*_pore_ and we extract its mean value. Fig. 5(a) shows results for increasing *n*_cyc_ in a gel with *ε* = 3.22ε*. As *n*_cyc_ increases, the distribution shifts to the right with a larger mean. In Fig. 5(b) illustrates the increasing behaviour of average poresize with *n*_cyc_ for (*ε*/ε*) = 1.45, 3.22. To provide a visual representation we present snapshots of slices through the gel in Fig. 5(c and d) for *n*_cyc_ = 0 and *n*_cyc_ = 1300, respectively. Initially, at *n*_cyc_ = 0, the system exhibits a smaller pore size. However, after multiple applied cycles, the larger pores tend to elongate, contributing to an overall increase in the average pore size. Some examples of elongated large pores are highlighted in Fig. 5(d): quantitative analysis of these (three-dimensional) features is challenging in general, but we intend to return to it in future work.

### 3.4. Summary

We find a coherent picture of the microscopic and mesoscopic structural changes in these cyclically sheared colloidal gels, both in the experiment and numerical simulations. The main effect is that shearing leads to a coarsening effect, where colloids' mean co-ordination number increases, as does the pore size of the gel. This can be rationalised in a simple way by the idea that the shearing injects energy and accelerates the random bond-breaking processes that are anyway occurring due to thermal fluctuations. In this way, it accelerates the natural aging/coarsening dynamics of the gel. However, the shearing procedure also introduces anisotropy to the system, which is apparent (for simulations) in the shape of the pores in the gel [Fig. 5].

Overall, the simulation model successfully captures the key features of structural changes across different length scales resulting from the repetitive mechanical deformation of the gel, including the microscopic structure of the arms. This complements previous results for effects of shear on the network topology, in which the internal structure of strands was not modelled in detail.^44,45^ In the next section, we use numerical simulations to investigate the mechanical response of the gel in more detail.

## 4 Results – mechanical properties (simulation)

So far, we considered cyclic shear of fixed amplitude *γ*_max_ = 0.04 in both experiment and simulation, and we analysed structural properties of the gel as a function of the number of cycles. In this section we exploit the ability of simulation to measure the time-dependent stress during cyclic shear. We also explore the dependence on the strain amplitude, revealing an interesting regime where the gel hardens under shearing.

### 4.1. Strain hardening before failure

#### 4.1.1. Hysteresis loops

**Fig. 6:**
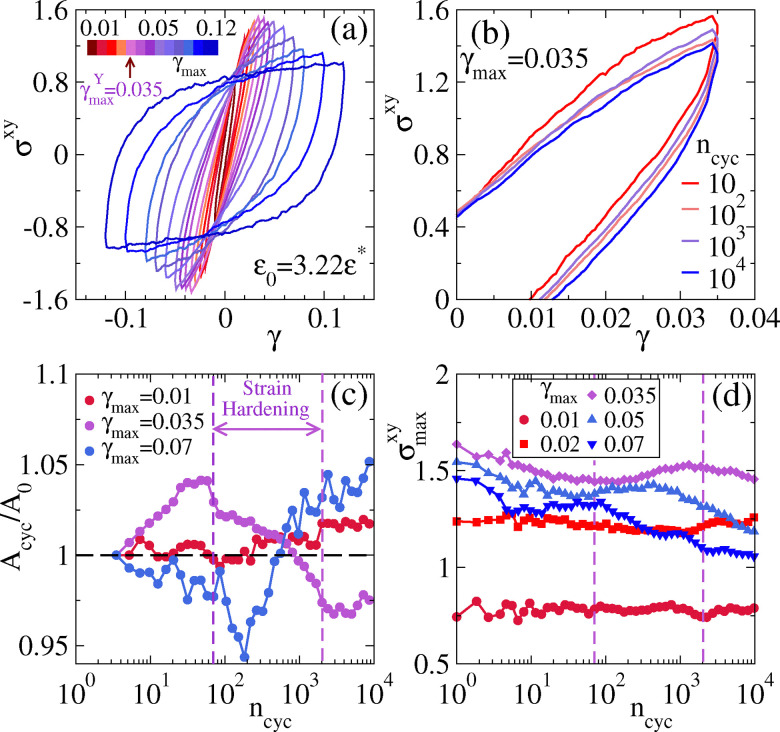
Cyclic shear for a simulated gel, interaction strength *ε* = 3.22ε*. (a) Average stress–strain curve over a strain cycle for several values of *γ*_max_ ranging from 0.02 to 0.12. (Data are averaged over the final 200 cycles, obtained from simulations of 10^4^ cycles.) (b) Zoomed view of stress–strain curves for *γ*_max_ = 0.035 at different values of *n*_cyc_. (c) The area enclosed by the stress–strain curve as a function of *n*_cyc_ (normalized with the value of initial cycle *A*_0_), for three different values of *γ*_max_. (d) Maximum stress *σ*^*xy*^_max_ at *γ*_*xy*_ = *γ*_max_ in a strain cycle against *n*_cyc_ for several *γ*_max_.

We consider gels with *ε* = 3.22ε*, as visualised in Fig. 5. Simulation results are shown in Fig. 6 for a range of *γ*_max_. (Note, the shear rate is held constant here and throughout at |*γ̇*| = 0.01*τ*_sim_^−1^, as stated in Section 2.2.) Specifically, Fig. 6(a) shows stress–strain curves, obtained from long simulations (*n*_cyc_ = 10^4^), averaged over the final 200 cycles. For very small *γ*_max_ and small shear rates *γ̇* one expects an elastic response with *σ*_*xy*_ ∝ *γ* and no hysteresis loop. We find here that some hysteresis is present even for *γ*_max_ = 0.01, which we attribute to the fact that the rate |*γ̇*| is not extremely small.

On increasing the amplitude *γ*_max_, the area of the hysteresis loop increases; the magnitude of the shear stress also depends non-trivially on *γ*_max_. This non-trivial dependence is associated with increasing dissipation, the onset of plastic deformation, and yielding. To characterise it, we write *σ*^*xy*^_max_ for the maximal stress during the cycle: this has a non-monotonic dependence on *γ*_max_.^34,71^ As a simple way to locate the crossover between elastic and plastic flow, we follow^34^ and identify the maximal value of *σ*^*xy*^_max_ with the transition to plastic flow; the associated shear amplitude is *γ*^*Y*^_max_. From Fig. 6(a) we identify *γ*^*Y*^_max_ = 0.0350 ± 0.0005. Alternative methods for characterising this crossover are discussed in ref. 97.

In Fig. 6(b) we show the evolution of the stress–strain loop as the number of shear cycles increases, for *γ*_max_ = 0.035, close to the yield strain.††Each loop is averaged over 10 cycles, they are not perfectly symmetric under inversion through the origin because the system is evolving structurally throughout the shearing process. Close inspection of the four curves shows that the dependence on *n*_cyc_ is non-monotonic. Fig. 6(c) plots the area of the loop (averaged over 10 consecutive cycles) as a function of *n*_cyc_, normalised by the value in the initial cycle. For *γ*_max_ = 0.035 the non-monotonic dependence is clear. We also define *σ*^*xy*^_max_ as the maximal stress during the cycle. This quantity is shown in Fig. 6(d), again showing non-monotonic dependence on *n*_cyc_ for *γ*_max_ = 0.035 (see also Fig. S9 of ref. 71 for an analogous effect in glasses).

These non-monotonic dependencies are associated with strain-hardening of the gel, as we now explain. Note first that for small strain amplitude *γ*_max_ = 0.01, the area and the maximal stress hardly depend on *n*_cyc_. This indicates that the gel is mostly responding elastically and the repeated shear cycles have little effect on its structure. For larger *γ*_max_ the overall trend is that the loop area increases and the maximal stress *σ*^*xy*^_max_ decreases. This indicates that the gel's response includes plastic rearrangements that disrupt its structure and lead to extra dissipation (larger loop area); the disruption to the structure also increases the compliance (reduces *σ*^*xy*^_max_). However, for *γ*_max_ = 0.035, the range labelled “strain hardening” in the figure is associated with a reduction in both the dissipation and the compliance. Similar effects are observed for other values of *γ*_max_ although the signature is most pronounced for *γ*_max_ ≈ *γ*^*Y*^_max_.

**Fig. 7:**
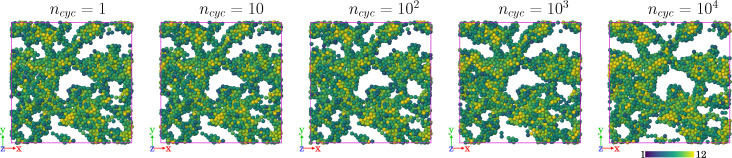
Snapshot of the gel structure as shearing takes place. The parameters are those of Fig. 6 [*ε* = 3.22ε*, *γ*_max_ = 0.035] so the system is strain-hardening from *n*_cyc_ = 10^2^–10^3^ and softening from 10^3^–10^4^, although the structural changes are subtle. The region shown has size *L* × *L* × *Z* with *Z* = 8*ℓ* (similar to Fig. 5), the colouring shows the co-ordination number (similar to Fig. 1).

Fig. 7 illustrates the corresponding evolution of the gel under shear. The main observation is that the changes are subtle, although the trends are consistent with Fig. 5 (which has slightly larger *γ*_max_ = 0.04). In particular, there is some coarsening of the structure with larger pores. The amorphous structure of the gel means that these small structural changes can nevertheless cause both strain softening and hardening behaviour, as a function of *n*_cyc_.

#### 4.1.2. Dynamic moduli

To probe further the strain-hardening phenomena, we investigate dynamic moduli (storage modulus *G*′, and loss modulus *G*′′). More specifically, we measure “nonlinear moduli” which incorporate the dependence of the stress response on the frequency and amplitude of an oscillatory (strain-controlled) shear. These quantities are familiar from studies of large amplitude oscillatory shear (LAOS).^98^ However, the standard theory is formulated for sinusoidal shear cycles, while our numerical data is obtained with a triangular (non-sinusoidal) waveform. To estimate the moduli in this case, we follow^99^ and reparameterise the time *t* in terms of a variable *s* = *s*(*t*) such that *γ*(*t*) = *γ*_max _sin(2π*s*(*t*)). We denote the time taken for a single shear cycle as *τ*_shear_ = *γ*_max_/(4*γ̇*), also note that *s*(*τ*_shear_) = 1 and define the corresponding angular frequency as *ω* = (2π/*τ*_shear_) = (8*πγ̇*/*γ*_max_).

**Fig. 8:**
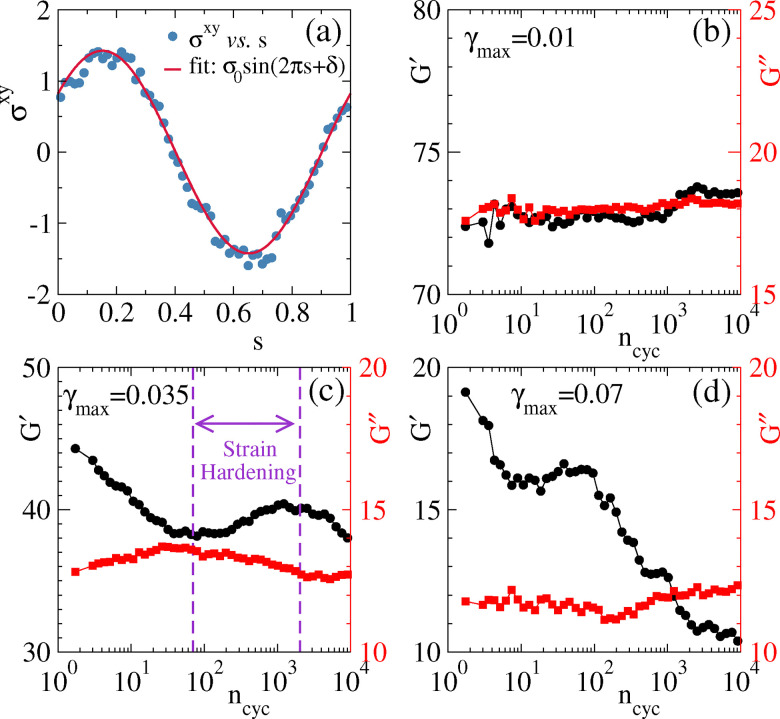
Estimated shear moduli for a simulated gel, based on shear protocols with different amplitudes (interaction strength *ε*_0_ = 3.22ε*). (a) Stress *σ*^*xy*^ as a function of rescaled time *s*. Solid line through the data points is a fit with *σ*^*xy*^ = *σ*_0_ sin(2π*s* + *δ*). Sample averaged storage modulus *G*′ and loss modulus (*G*′′) as a function of strain cycle for (b) *γ*_max_ = 0.01, (c) *γ*_max_ = 0.035, and (d) *γ*_max_ = 0.07.

The stress profile for any shear cycle can then be fitted as a function of *s*, as *σ*(*s*) = *σ*_0_ sin(2π*s* + *δ*) where the amplitude *σ*_0_ and phase lag *δ* are fitting parameters. An example fit is shown in Fig. 8(a). This allows nonlinear moduli *G*′ and *G*′′ to be extracted from the formula6
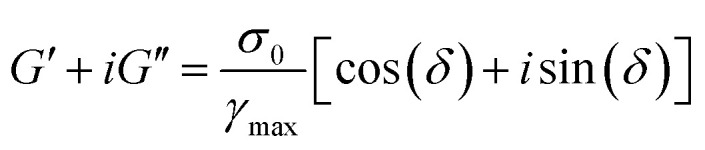
where *σ*_0_,*δ* are obtained from the fit, while the amplitude *γ*_max_ is set by the underlying shear cycle. Since the gel is hardening as the shear accumulates, we perform this fitting separately for each individual cycle. We average the resulting *σ*_0_,*δ* over many independent samples.‡‡We verified that similar results are obtained by averaging the time-dependent stress over the samples and then fitting the average.

The resulting moduli depend on (*ω*,*γ*_max_,*n*_cyc_); recall that *γ̇* is fixed throughout this study so we study *G*′, *G*′′ as functions of (*n*_cyc_,*γ*_max_). Results are shown in Fig. 8(b–d) for different values of strain amplitude. In each case we measure how the moduli change as the oscillatory shear accumulates (increasing *n*_cyc_). We discuss the different amplitudes in turn.

For small amplitude *γ*_max_ = 0.01 [Fig. 8(b)], both *G*′ and *G*′′ remain almost constant. For larger amplitude *γ*_max_ = 0.035 (close to the yield strain), Fig. 8(c), shows more interesting behaviour, in that both *G*′ and *G*′′ are non-monotonic functions of the number of cycles *n*_cyc_. An initial decrease in *G*′ and a corresponding increase in *G*′′ indicate gel softening. At intermediate cycle number, however *G*′ begins to rise while *G*′′ decreases, indicating hardening of the gel. Notably, this transition region aligns with our earlier observations – specifically, the reduction in the stress–strain loop area and the increase maximum stress as shown in Fig. 6(c and d).

For a yet larger strain amplitude *γ*_max_ = 0.07 [Fig. 8(d)], a similar hardening behavior is observed, but it is restricted to a narrower range of cycles (*n*_cyc_ = 10–100). Beyond this regime the gel exhibits liquid-like characteristics, as indicated by *G*′ < *G*′′ for *n*_cyc_ > 10^3^.

#### 4.1.3. Physical interpretation

Fig. 6 and 8 demonstrate both thixotropy and strain hardening, for appropriate ranges of *γ*_max_ and *n*_cyc_. Physically, thixotropy is expected in situations where shearing disrupts the gel network, for example by breaking strands and opening up larger pores (recall Section 3). On the other hand, strain-hardening can be rationalised because shearing tends to anneal the local structure, leading to higher co-ordination and lower energy clusters (as observed by the TCC, for example).

The competition between these effects leads to a complicated phenomenology where strain hardening is strongest for intermediate *γ*_max_ close to *γ*^*Y*^_max_ (see also ref. 54); it is also notable that the value of *γ*_max_ affects the range of *n*_cyc_ over which strain hardening is observed. Future work should investigate the interplay between structure and dynamics in more detail, in order to predict and control whether shearing leads to more or less compliant gels.

Taken together, these results complement previous works that demonstrated strain-hardening in other gel models, which had different microscopic interactions (and where thermal fluctuations were neglected).^44,45^

### 4.2. Delayed failure time of cyclically sheared gel

Given the strain-hardening behaviour demonstrated above, one may expect that the resulting gels are also more stable: we now demonstrate that this is indeed the case. We simulated creep dynamics at constant stress, following.^84^ The initial conditions for this procedure are gel configurations that have been cyclically sheared for *n*_cyc_ strain cycles. We then impose a constant (non-dimensionalised) shear stress *σ*_0_ in the *xy* plane, allowing flow along the *x*-direction with Lees–Edwards boundary conditions (which is the same geometry used for the cyclic shear). We take *σ*_0_ = 0.8, this value is chosen such that the system undergoes creeping flow and then fails within our computational time window.§§See ref. 84 for further discussion of the *σ*_0_-dependence: we would expect yielding to be similar at smaller *σ*_0_, if sufficiently long simulations were performed. The behaviour for larger *σ*_0_ is different because the system yields almost immediately.

**Fig. 9:**
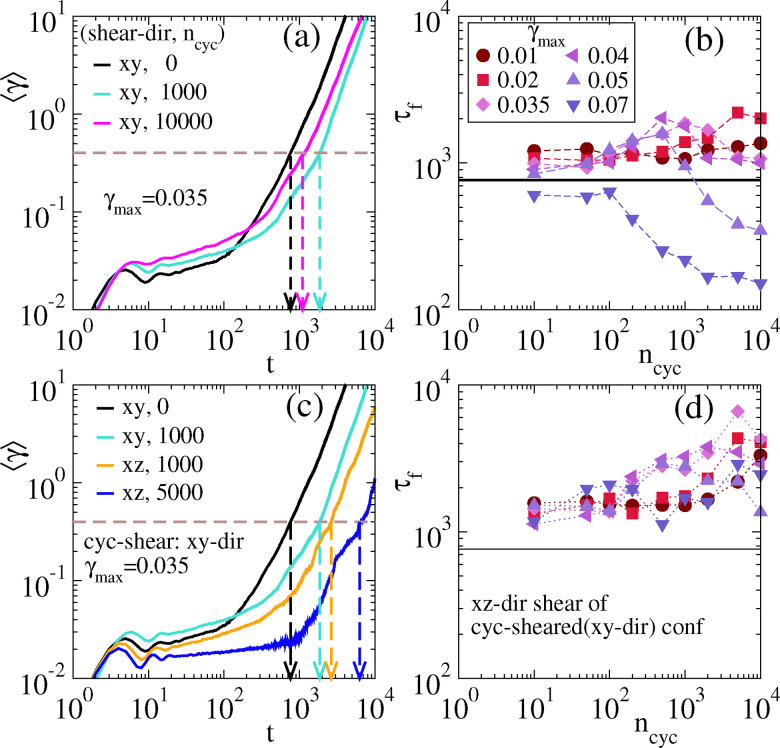
Creep Simulation, interaction strength *ε*_0_ = 3.22ε* (a) Average strain against time for different values of *n*_cyc_ = 0, 10^3^, 10^4^. The dashed horizontal line represents *γ* = 0.4, which is taken as the critical value of strain beyond which system starts to flow. Vertical dashed lines indicate the failure time *τ*_f_ for each case. (b) Failure time *τ*_f_ as a function of *n*_cyc_ for different *γ*_max_. (c) Strain against time for shear applied to different directions and (d) the corresponding failure time as a function of *n*_cyc_.

We measure the strain during this creep flow: its average behaviour is shown in Fig. 9(a), for samples that have been through *n*_cyc_ = 0, 10^3^, 10^4^ shear cycles, with *γ*_max_ = 0.035. The time-dependent strain has an initial elastic branch followed by a plateau, creeping flow, and eventually failure. The cyclic shear increases the height of the plateau: its value is comparable with *γ*_max_ indicating that the structure of the gel has rearranged during cyclic shear so that it can accommodate this strain.^100^

We define the failure time *τ*_f_ of the gel as the time for 〈*γ*〉 to reach a threshold γ* = 0.4, as explained in ref. 84. From Fig. 9(a), the failure time is largest for *n*_cyc_ = 10^3^ cycles, after which it decreases again. This is consistent with Fig. 6, which indicates that the strain-hardening period ends at around *n*_cyc_ = 2 × 10^3^. That is, strain hardening is also accompanied by an increased failure time.

We performed creep simulations for a range of *γ*_max_ and *n*_cyc_, from which we computed the failure times. Results are collated in Fig. 9(b), which we compare with “pristine” gels (*n*_cyc_ = 0). For *γ*_max_ = 0.035, strain hardening persists up to *n*_cyc_ = 10^3^, beyond which the material begins to soften again. This reinforces the conclusion of Fig. 9(a). The same characteristic non-monotonicity is also observed for other cyclic shear amplitudes in the range 0.02 ≤ *γ*_max_ ≤ 0.05, and the ranges of *n*_cyc_ over which this is observed mirror the ranges of strain hardening in Fig. 6(d). For small amplitude, *γ*_max_ = 0.01, the failure time for *n*_cyc_ = 10 is enhanced with respect to the pristine gel. After this, *τ*_f_ is weakly affected by further shearing. This mirrors the small structural change due to such cyclic shear [recall Fig. 6]. For large amplitude *γ*_max_ = 0.07 the failure time is reduced by the cyclic shear, presumably because this significantly disrupts the gel network.

These moderate enhancements of *τ*_f_ by cyclic shear (up to a factor of 3) are affected by two competing processes. The agitation by shearing promotes coarsening of the gel structure, and activates relaxation processes that allow it to descend down the energy landscape, as occurs in physical aging. This tends to increase the failure time *τ*_f_. At the same time, the repeated cyclic strain imprints an anisotropic memory on the gel structure: this may predispose it to flow along the direction of the shear, reducing *τ*_f_. (This process may be understood through the framework of fatigue failure,^101,102^ where damage accumulates progressively until the system ultimately fails.).

To disentangle these effects, we simulated creeping flow with constant stress applied in the *xz* direction (the flow is still along *x* but the gradient is now in *z*). Results for *γ*_max_ = 0.035 are shown in Fig. 9(c). Comparing with Fig. 9(a), one sees that the enhancements of *τ*_f_ are much stronger when the creep flow is not in the same plane as the cyclic shear. Our interpretation is that the increase of *τ*_f_ due to coarsening and aging effects is dominating this response due to *xz*-stress, because memory effects due to *xy*-shear are less relevant in this geometry. That is, changing the direction of the shear stress in creep simulations separates the coarsening/shearing effects from the damage accumulation due to previous cyclic shear in the *xy* plane. Surprisingly, the increase of *τ*_f_ with *n*_cyc_ continues even up to *n*_cyc_ = 5000, at which time Fig. 6(d) suggests that the system has entered the shear-softening regime. This result further illustrates the complex dependence of gel structure and response on its mechanical history.

Fig. 9(d) plots the failure times for creeping flow with constant *xz*-stress, showing that *τ*_f_ is generically increased by shearing, regardless of whether the system is in the strain-hardening or softening regime. It seems that the softening effect at large *n*_cyc_ in Fig. 6(c and d) is an anisotropic effect, as may be expected from the mechanical protocol used. Given the strong anisotropy of the response [visible as differences between Fig. 9(b and d)], it would be interesting to explore in more detail the effects of different cyclic shear protocols on failure. (For example, combining cyclic shears in more than one plane^59^ might generate more isotropic structures.)

## 5. Discussion

Colloidal gels are arrested far from equilibrium, so it is expected that their properties are affected by their mechanical history, including cyclic shear. However, it remains challenging to predict these effects and exploit them for material design: materials' history-dependence is complex in general and there may be competing effects. For example the shearing may break gel strands, but it also leads to coarsening and hence to thicker strands. In this situation, particle-resolved experiments and simulations offer valuable opportunities to disentangle different behaviors and to understand their microscopic mechanisms.

We have made progress in this direction, showing in both experiments and simulations that shearing leads to coarsening of the gel network and to denser microscopic packing within the strands and local structure indicating states deeper in the energy landscape. Consistent with recent work on glasses, this illustrates the general principle that shearing offers a mechanism for accelerating the progress of materials towards lower free-energy states. We also showed that these structural changes also affect material responses like compliance and stability.

Previous work on sheared gels^55,63,64^ has highlighted the role of the Mason number Mn as a measure of whether the shear stress is strong enough to break interparticle bonds (see also the depletion Peclet number of ref. 47). Following^63^ we define Mn = 3π*η*_s_*ℓ*^3^*γ̇q*/(2*ε*) = 12*γ̇τ*_B_*q*/*ε*_0_ (recall that *q* is the interaction range measured in units of the diameter, it appears because a typical interparticle force is the well-depth *ε* divided by the range). As already discussed, the dimensionless shear rates for the experiments (Table 1) are much larger than those of the simulations, which is fixed throughout at *γ̇τ*_B_ ≈ 0.004 (Section 2.2). This leads to Mn ∼ 10^−4^ for the simulations, but Mn ∼ 1 for the experiments. At face value, this suggests that the two systems might be in quite different regimes, despite their similar behaviour.

However, we emphasize that we have performed cyclic shear with relatively small strains (a few percent), contrary to^55,63,64^ which considered either steady flow or shear cessation, where the strains are much larger. Our interpretation is that the amplitude *γ*_max_ of the cyclic shear strain is controlling the behaviour, while the shear rate (hence also the Mason number) plays a secondary role. Physically: even if Mn is larger than 1, the system can accommodate small shear strains without bonds breaking. This interpretation is supported by Fig. 6 in which increasing *γ*_max_ causes a qualitative change in behaviour, while Mn remains constant. In this sense the regime studied here is different to that of ref. 64. For even larger Mn one may expect that the shear rate becomes relevant again but this situation is not considered here. This issue could be usefully addressed in future work.

More generally, our understanding of gel rheology will inform future work that aims to predict effects of mechanical processing, and use them to design material properties. Theoretical insights would be very valuable in this endeavour, because the multi-scale structure of gels means that many colloidal particles have to be simulated (or tracked) in order to model the behaviour of a single gel strand, while much of the important physics is happening on larger length scales, *via* properties of the gel network. This points towards the development of simplified meso-scale models, although there are significant couplings between microscopic structure and the responses of gel strands, which indicate that these theoretical models should not be *too* simple. In any case, we are optimistic that prediction of history-dependent properties can be improved beyond the current state-of-the-art, *via* a combined approach based on theory, experiment, and computer simulations.

## Conflicts of interest

There are no conflicts to declare.

## Data Availability

The data in this article are available at https://doi.org/10.17863/CAM.119276. This includes the data that underlies the figures, the custom analysis scripts, and input parameters used to run molecular dynamics simulations *via* LAMMPS.
